# The pattern and magnitude of T cell subsets reconstitution during ten years of ART with viral suppression in HIV-infected patients

**DOI:** 10.18632/aging.204416

**Published:** 2022-12-09

**Authors:** Lianfeng Lu, Xiaodi Li, Xiaosheng Liu, Zhifeng Qiu, Yang Han, Xiaojing Song, Yanling Li, Xiaoxia Li, Wei Cao, Wei Lv, Zhihui Dou, Taisheng Li

**Affiliations:** 1 Department of Infectious Diseases, Peking Union Medical College Hospital, Peking Union Medical College and Chinese Academy of Medical Sciences, Beijing, China; 2 Tsinghua-Peking Center for Life Sciences, Beijing, China; 3 National Center for AIDS/STD Control and Prevention, Chinese Center for Disease Control and Prevention, Beijing, China; 4 State Key Laboratory of Complex Severe and Rare Diseases, Peking Union Medical College Hospital, Chinese Academy of Medical Science and Peking Union Medical College, Beijing, China

**Keywords:** HIV, antiretroviral treatment, CD4+T cell, CD4/CD8, T cell subsets, immune reconstitution

## Abstract

Background: The extent of immune reconstitution in human immunodeficiency virus (HIV) infected persons receiving long-term antiretroviral therapy (ART) with controlled viral load has been controversial. We studied the extent and speed of T cell subsets retrieval after long-term antiretroviral treatment.

Methods: 662 HIV-infected patients followed at least 2 years whose plasma HIV-1 RNA load <50 copies/mL were evaluated for longitudinal and functional phenotypic indices of immune restoration. Determinants of change in magnitude and importance of recovery have been evaluated using mixed linear regression models.

Results: Almost all robust immune restorations achieved occurred after 2–3 years of ART. The median CD4 lymphocyte count increased 449 cells/μl (IQR 303–604) from 226 cells/μl (IQR 83–336) at baseline during the third year (*P* < 0.001); CD4+T lymphocyte rises during the sixth and tenth years were not significant. Naive and memory CD4+T cells‘reconstitution occurred in the sixth and eighth years of ART but no significant change thereafter. The change of CD45RA+Naïve and CD45RA-memory CD4+T cell reconstitution is different in baseline CD4+T cell counts <100 cells/μl group and in baseline CD4+T cell counts >100 cells/μl group. Activation antigen expression (CD38 or HLA-DR) on CD8 lymphocytes declined mostly during the first till second year, and after 4 years, activation antigen expression on patient lymphocytes showed no significant change. The proportion of CD4 cells expressing CD28 climbed during the first years and reached normal levels in the second year.

Conclusions: Immune restoration was dependent on the capacity of immune system during the first 2–3 year of ART. But the significant change of CD4 and compartments of CD4+T cells could persist until 6–8 years. The pattern of CD38+CD8+, HLA-DR+CD8+, CD28+CD4+ T cells could quickly return to normal level and no significant change after sufficient time of ART. In general, the immune response compared to the baseline status may be the overall effect from the age and time of antiretroviral treatment.

## INTRODUCTION

Infection with the human immunodeficiency virus (HIV) will lead to loss of CD4+T lymphocytes, increasing susceptibility to severe immunodeficiency, opportunistic infection and even death. The number of people infected with HIV is continually increasing worldwide and patients receiving antiretroviral therapy (ART) is climbing to 27.5 million worldwide, according to WHO reports up to 2020 [1]. The immune system of HIV-infected patients recovered after ART, which is generally evaluated by the increased number of CD4+T lymphocytes after undetectable viral load [2–5].

It has been acknowledged that CD4+T cells will increase over a period in HIV-infected patients with viral suppressed. However, the data were ambiguous with respect to the increasing period and degree of CD4+T cells. It is reported that CD4+ T cells climbed quickly within the 1–2 years of treatment, but slowly increased to 4–8years, regardless of baseline CD4+ T cell value [6–9], or weather CD4 count stabilizes. Most analyses on CD4 counts have set limit of times when plasma HIV-1 RNA (pRNA) was suppressed, e.g., to <400 copies/ml or <1000 copies/ml [7, 8], while the lowest detection limit of HIV detection has become less than 20 copies/ml. Nowadays, the characteristics of immune system response in HIV patients that meet the lower limits of what is considered viral suppression are likely different from many years ago, leading to possible selection biases. As a result, we still require up-to-date data for the CD4+T cell count increment after restricted viral suppression.

On the other hand, the comprehensive assessment of the immune response should cover up the increased number of CD4+ T cells and concentrate on the other T cell phenotype like T cell activation related to virus fluctuate during ART treatment. However, only a small number of investigations with a limited sample size have shown the kinetics of T cell subsets such as CD4+CD28+T cells and CD4+CD45RA+T cells after short-term follow-up [10–12]. Some researchers have focused on the sub-population of T lymphocytes in children [13]. The characteristics of this research included a viral load limit detected below 400 copies/ml, patients enrolled having viral load higher than 400 copies/ml and a sample size of less than 50. In conclusion, these studies have not provided a complete explanation of the change in the T cell subpopulation after viral suppression according to current guidelines [14].

Overall, we aim to show the immune system response after long-term (10-year) ART with undetectable viral load in our patients. The assessment of immune system will include CD4+T cell counts, CD8 cell counts, CD4/CD8 ratio, and the number of naive CD45RA+CD4+T cells and memory CD45RA-CD4+T cells, the CD38+CD8+T cells, CD8+HLA-DR+T cells and CD28 expression on CD4+ and CD8+T cells in the peripheral blood. In this study, we analyze the data by fitting the kinetics of selected parameters and related risk factors with mixed linear model in patients who received ART at least two years and maintained viral load <50 copies/ml.

## RESULTS

### Clinical and immunology characteristics at baseline

In 662 HIV- infected patients, 88.2% (584) of them were male (Table 1). The mean age was 36.4 ± 11.2 years. The sexual transmission was the most frequent in transmission routes, especially in patients having sex between men (63.0%). 65.1% of patients has one or more signs or symptoms of HIV infection. 212 participants (32.0%) had an opportunistic infection and 43 (6.5%) of enrolled patients were HBsAg positive or 24 (3.6%) were HCV-Ab positive. Mean log_10_ (viral load) of these patients was 4.74 ± 0.77. 36.3% of patients’ baseline viral load is higher than 10^5^ cps/ml, which represents a relatively high level of viral load. 225 patients (34.0%) did not achieve viral suppression at the first six months, most patients (74.8%) initiated ART with two Nucleoside Reverse Transcriptase Inhibitor (NRTIs) with non-Nucleoside Reverse Transcriptase Inhibitors (NNRTIs). The median follow-up time was 5.67 (3.17, 8.33) years.

**Table 1:** Baseline characteristic of patients and status after follow-up period.

**Time after follow-up**					
**Characteristics**	**Baseline (*n* = 662)**	**1-year (*n* = 652)**	**3-year (*n* = 515)**	**5-year (*n* = 342)**	**10-year (*n* = 89)**
Age, (year, mean ± SD)	36.4 ± 11.2	37.3 ± 11.2	39.4 ± 10.9	41.2 ± 10.9	48.3 ± 9.8
Age (*n*, %)					
> = 50	90 (13.6)	93 (14.2)	88 (17.0)	72 (17.6)	33 (33.7)
Male, *n* (%)	584 (88.2)	574 (88.0)	455 (77.8)	297 (86.8)	69 (69.7)
Route of transmission, *n* (%)
Homosexual	417 (63.0)				
Heterosexual	103 (15.6)	NA	NA	NA	NA
Blood	53 (8.0)				
Unclear/others	89 (13.4)				
Opportunistic infection, *n* (%)	212 (32.0)	NA	NA	NA	NA
HBsAg (+), *n* (%)	43 (6.5)	NA	NA	NA	NA
HCV-Ab (+), *n* (%)	24 (3.6)	NA	NA	NA	NA
Baseline ART regimen, *n* (%)^†^		NA	NA	NA	NA
2 NRTIs + NNRTI	495 (74.8)				
2 NRTIs + PI	45 (6.8)				
2 NRTIs + INRTI	73 (11.0)				
Other	49 (7.4)				
Plasma viral load (log_10_copies/mL)	4.74 ± 0.77	NA	NA	NA	NA
<5	407 (61.5)				
≥5	240 (36.3)				
Mean time suppressed to VL <50 cps/ml (month)	9.9 ± 17.2	NA	10.6 ± 18.0	12.9 ± 20.9	23.5 ± 32.2
>6 month	225 (34.0)		216 (36.7)	174 (42.6)	67 (67.7)
CD3+CD4+T counts (cells/μl) Ref (561–1137)	226 (83,336)	376 (244, 533)	449 (303, 604)	468 (352,623)	500 (389, 653)
~49	124 (18.7)	4 (0.6)	4 (0.8)	2 (0.6)	1 (1.1)
50~99	56 (8.5)	27 (4.1)	4 (0.8)	2 (0.6)	0 (0.0)
100–199	122 (18.4)	83 (12.7)	35 (6.8)	10 (2.9)	1 (1.1)
200–349	215 (32.5)	183 (28.1)	125 (24.3)	68 (19.9)	14 (15.7)
350–499	96 (14.5)	168 (25.8)	138 (26.8)	109 (31.9)	28 (31.5)
≥500	49 (7.4)	187 (28.7)	209 (40.5)	151 (44.2)	45 (50.6)
CD3+CD8+T counts(cells/μl) Ref (404–754)	789 (532,1045)	732 (548, 969)	699 (527, 925)	666 (518,863)	647 (515, 843)
~499	147 (22.2)	122 (18.7)	111 (21.6)	82 (24.0)	20 (22.7)
500–999	300 (45.3)	380 (58.4)	297 (57.8)	208 (60.8)	55 (62.5)
1000–1499	151 (22.8)	115 (17.7)	93 (18.1)	41 (12.0)	8 (9.1)
≥1500	64 (9.7)	34 (5.2)	13 (2.5)	11 (3.2)	5 (5.7)
CD4+CD45RA-/CD4+T (%) Ref (45.6–68.4)	71.6 (61.5, 84.2)	69.9 (60.0, 80.4)	68.6 (59.3, 79.0)	70.9 (61.5, 79.8)	74.5 (66.4, 83.9)
CD4+CD45RA-T count (cells/μl) Ref (283–683)	150 (73, 226)	249 (175, 348)	300 (217, 390)	326 (246,417)	374 (290, 456)
CD4+CD45RA+/CD4+T (%) Ref (31.6–54.4)	28.6 (15.9, 38.5)	30.1 (19.7, 40.0)	31.3 (21.0, 40.7)	29.2 (20.2,38.6)	25.5 (16.1, 33.7)
CD4+CD45RA+/CD4+T Ref (206–530)	62 (14,117)	112 (50, 192)	135 (69, 218)	133 (79, 210)	120 (66, 203)
CD4+CD28+/CD4+T (%) Ref (85.0–100.0)	89.2 (75.0, 95.5)	92.2 (82.8, 96.8)	93.3 (86.5, 97.4)	93.9 (86.9, 97.5)	93.2 (86.9, 98.0)
CD8+CD28+/CD8+T (%) Ref (37.2–60.4)	30.5 (21.7, 40.1)	40.8 (31.5, 52.7)	48.5 (38.6, 60.1)	52.3 (42.0, 63.2)	59.6 (44.0, 66.9)
CD8+HLA-DR+/CD8+T (%) Ref (6.3–23.8)	63.2 (48.3, 74.2)	40.3 (29.4, 52.9)	37.3 (26.8, 48.9)	35.2 (25.3, 45.6)	34.9 (27.2, 49.4)
CD8+CD38+/CD8+T (%) Ref (32.4–57.4)	79.8 (68.5, 89.3)	51.6 (37.4, 65.4)	44.7 (32.1, 57.3)	41.6 (30.2, 54.1)	31.0 (21.5, 44.8)
CD4+/CD8+T Ref (0.95–2.13)	0.25 (0.12, 0.40)	0.53 (0.28, 0.80)	0.66 (0.41, 0.92)	0.72 (0.47, 1.02)	0.76 (0.57, 1.04)
> = 1.0 (*n*, %)	8 (1.2)	92 (14.1)	102 (19.7)	91 (26.5)	28 (31.5)

Abbreviations: ^†^NRTIs: nucleoside reverse transcriptase inhibitor; NNRTI: Non-nucleoside reverse transcriptase inhibitor; INSTI: integrase strand transfer inhibitor; PI: protease inhibitor. Ref: Reference range based on healthy controls.

The demographic and T cell subpopulation characteristics of patients were presented in Table 1, based on their follow-up periods (Y = 1 (*n* = 652), Y = 3 (*n* = 515), Y = 5 (*n* = 342), and Y = 10 (*n* = 89)). In enrolled HIV-infected patients, the median number of CD4+T cells and median number of CD8+T cells at baseline were 226 cells/μl (interquartile range, hereafter IQR 83–336) and 789 cells/μl (IQR 532–1045), respectively. The median percentages of naive CD4+T and median percentages of memory CD4+T cells at baseline in overall patients were 28.6% (15.9%, 38.5%) and 71.6% (61.5%, 84.2%), respectively. Median baseline percentages of CD28+CD4+T cells and CD28+CD8+T cells, CD38+CD8+T cells, HLA-DR+CD8+T cells, CD4+/CD8+T in overall patients were abnormal than the reference range (shown in Table 1). For patients followed at least 3-year, 209 (40.5%) patients achieved CD4+T cells >500 cells/μl, the percentage climbed at 44.2%, and 50.6% in patients followed for 5 and 10 years. Also, there were 7.4% patients whose baseline CD4+T cell over 500 cells/μl. Besides, the percentage of CD8+T cells in 500–999 cells/μl and 1000–1499 cells/μl changed from 45.3%, 22.8% till 62.5%, 9.1% after 10-year of ART. Whereas, the percentage of CD4/CD8> = 1 kept increasing from 1.2% to 31.5% until 10 years.

### Dynamics of absolute counts of CD4+ T cell, naive CD4+ T cells, and memory CD4+T cells

**Figure 1:**
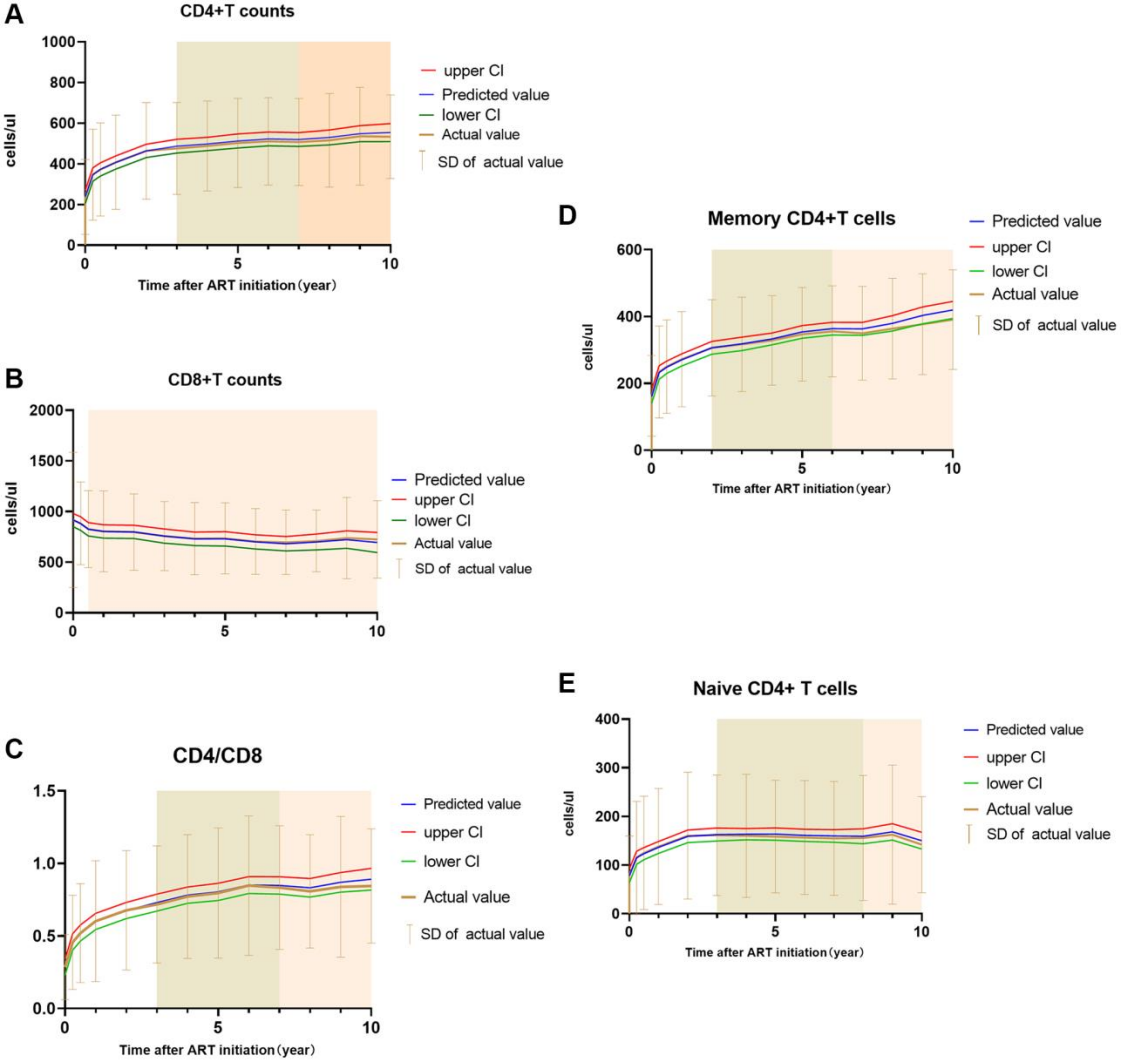
Fitted curve of estimated trends and calculated mean with SD of the count CD4+T cell (**A**), CD8+T cell (**B**), CD4/CD8 (**C**), Memory CD4+T cell (**D**) and Naïve CD4+T cell (**E**). Notes: predicted value means the predicted indicators calculated by the mixed linear model used in the article, upper CI and lower CI means the upper confidence interval and lower confidence interval of predicted value. Actual value and SD were calculated from original data. The green shade represented a significant change during 2–3 year interval, the pink shade represented no significant change during that time.

The median number of CD4+T cell counts was 226 cells/μl (IQR 83–336) before treatment and increased to 449 cells/μl (IQR 303–604) after 3-year of ART (Table 1). Through fitting the increasing curve of CD4+T cells, an initial surge in CD4+T cell counts within 3 months was evident, but relatively slower accumulation in CD4+T cell count until the fourth year after treatment (Table 2 and Figure 1A). After the fourth year of steady increment, the significant change period of CD4+T cell counts prolonged to 2-year until the seventh year while there was no significant difference of CD4+T cells between the next three year (Figure 1A and Supplementary Table 1). Given the covariate factors that may affect the increase in CD4+T cell counts, it was showed that the ART scheme including Protease Inhibitors (PI), Integrase Inhibitor (INSTI) and the CD4+T cell counts (>50 cells/μl) at baseline were beneficial factors.

**Table 2:** The influencing factors of CD4+T cell count, Naïve CD4+ T cell and Memory CD4+T cell dynamics with mixed linear model.

	**CD4+ T cell count**		**Naïve CD4+ T cell**		**Memory CD4+ T cell**	
	**Unadjusted Est. Coef (SE)^†^**	**Adjusted Est. Coef (SE)^†^**	**Unadjusted Est. Coef (SE.)^†^**	**Adjusted Est. Coef (SE.)^†^**	**Unadjusted Est. Coef (SE)^†^**	**Adjusted Est. Coef (SE)^†^**
Age						
35–49	−29 (17.4)	11 (10.5)	−33 (8.1)	−15 (5.5)	5 (10.5)	20 (7.3)^‡^
>50	−35 (24)	−21 (14.7)	−55 (11.3)	−16 (7.6)^‡^	19 (14.6)	51 (10.3) ^‡^
18–35	0					
Gender						
Female	−30 (24.2)	−17 (14.5)	−21 (11.5)	4 (7.7)	−5 (14.5)	1.4 (10.1)
Male	0					
CD4 count at baseline (cell/μl)						
50–100	44 (25.3)	49 (19.4)^‡^	NA	NA	NA	NA
100–200	119 (20.2)	128 (15.3^‡^	NA	NA	NA	NA
>200	308 (16.4)	329 (12.6)^‡^	NA	NA	NA	NA
<50	0					
Years after ART initiation (years)						
0.25	109 (4.6)	108 (5.1)^‡*^	37 (2.5)	33 (3.3)^‡*^	72 (3.5)	72 (3.8)^‡*^
0.5	133 (6.2)	133 (7.1)^‡*^	46 (3.3)	40 (3.7)^‡*^	87 (4.6)	87 (5.3)^‡*^
1	169 (7.3)	167 (8.3)^‡*^	59 (3.9)	51 (4.0)^‡*^	110 (5.3)	110 (6.2)^‡*^
2	225 (8.2)	224 (9.1)^‡*^	82 (4.3)	73 (4.1)^‡*^	145 (5.8)	146 (6.8)^‡*^
3	255 (9.1)	253 (9.8)^‡*^	93 (4.8)	87 (4.5)^‡^	160 (6.4)	162 (7.3)^‡*^
4	275 (9.9)	274 (10.5)^‡^	99 (5.3)	87 (4.8)^‡^	174 (7.0)	177 (7.7)^‡^
5	291 (11.1)	292 (11.2)^‡^	100 (5.8)	90 (5.0)^‡^	192 (7.7)	197 (8.2)^‡^
6	312 (11.8)	315 (11.9)^‡^	103 (6.3)	95 (5.3)^‡^	208 (8.2)	214 (8.7)^‡^
7	316 (12.9)	322 (12.7)^‡^	105 (6.8)	102 (5.8)^‡^	204 (8.9)	213 (9.3)^‡^
8	334 (14.2)	347 (13.7)^‡*^	112 (7.5)	102 (5.8)^‡*^	220 (9.8)	232 (10.0)^‡^
9	357 (16.1)	377 (15.3)^‡*^	118 (8.5)	110 (6.5)^‡*^	234 (11.0)	249 (11.2)^‡*^
10	350 (18.5)	380 (17.4)^‡*^	105 (9.8)	109 (7.3)^‡^	239 (12.7)	258 (12.7)^‡*^
0						
ART regimen						
2NRTIs+PI	84 (31.8)	105 (19.2)^‡^	42 (15.2)	37 (9.9)^‡^	42 (19.2)	20 (13.4)
2NRTIs+INSTI	4 (26.8)	68 (16.8)^‡^	−1 (12.8)	15 (8.1)	3 (16.4)	28 (11.7)^‡^
Others	55 (30.1)	63 (18.0)^‡^	25.6 (14.36)	13 (9.4)	23 (18.1)	7 (12.5)
2NRTIs+NNRTI	0					
VL<50cps/ml						
No	84 (4.8)	1 (5.6)	32 (2.5)	9 (3.0)^‡^	59 (3.6)	0 (4.2)
Yes	0					
Baseline naïve+T cell increase per 50/ul	NA	NA		68 (2.0)		55 (2.7)

Abbreviations: ^†^Est. Coef.: estimated coefficient; SE.: standard error ^‡^Between group comparisons of the difference of values at particular time points and the baseline value, *P*-Value <0.05; ^*^Adjusted multiple comparisons when compared with indicators at 5-year of ART, *P*-Value < 0.05.

Absolute CD45RA+Naïve CD4+T cells started from 62 cells/μl (IQR, 14–117) at baseline to 135 cells/μl (IQR 69–218), 133 cells/μl (IQR,79,210), 130 cells/μl (IQR,76,198), 120 cells/μl (IQR,66,203) after 3 years, 5 years, 8 years and 10 years of ART, respectively (Table 1). A significant climbing on the Naïve CD4+T cells in HIV patients in every-year at first 3-year of ART but every two year of significant change for 3–8 year (Figure 1B).

The number of absolute CD45RA-Memory CD4+T cells was 150 cells/μl (IQR, 73–226) before treatment. After 3 years,5 years, 8 years, and 10 years of ART, it increased to a median of 300 cells/μl (IQR, 217–390), 326 cells/μl (IQR, 246, 417), 349 cells/μl (IQR, 265–441), 374 cells/μl (IQR, 290–456), respectively (Table 1). Unlike CD45RA+ naïve CD4+T cells, there was a significant change in CD45RA- CD4+T cells within 3 months of treatment initiation, but there was no evident change between first 3-month and 6-month. The significant increasing span went from one year during 0–3 years after ART and two years in 3–6 years (Figure 1C), and there was no significant increase thereafter through multiple comparisons (Seen in Table 2 and Supplementary Table 2).

**Figure 2:**
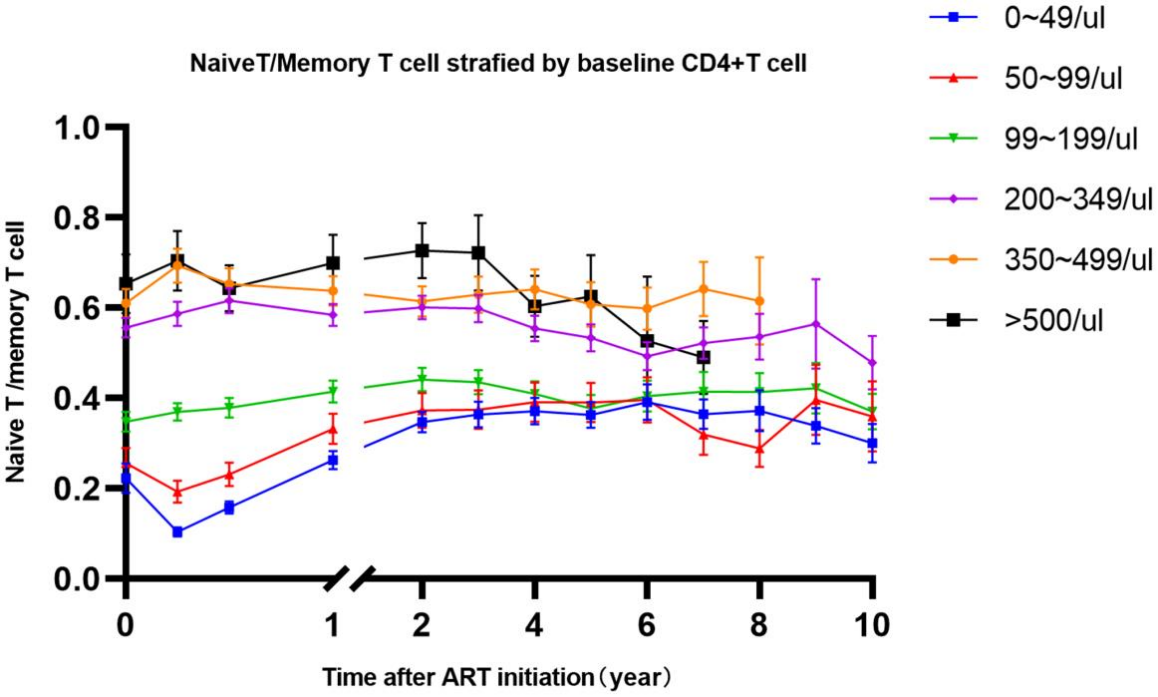
**The trends of naïve/memory T cell change after 10-year of ART stratified by baseline CD4+T cells.** Notes: Different color represents different level of baseline CD4+T cell counts, which shown in top right corner.

In the mixed linear model, no significance of naïve and memory CD4+T cell counts between gender was found. Age was independently inversely linked to the CD45RA-Memory and CD45RA+CD4 T cell counts. CD4+CD45RA-T cells increased with age, while CD45RA+Naïve CD4+T cell counts decreased with age group. ART regimens (2NRTIs+PI and 2NRTIs+INSTI) have marked changes of naïve CD4+T cell and Memory CD4+T cells, which are consistent with results from CD4+T cell counts (Table 2). Since CD4+T cell counts were composed of CD4+naïve and CD4+memory T cells, it is unreasonable to put this indicator in the mixed model, so we calculated the trends of naïve and memory T cell ratio stratified by baseline CD4+T cell counts. It is shown that the kinetics of naïve/memory CD4+T cells decreased at first three months in patients whose CD4+T cell counts <100 cells/μl. However, in patients with CD4+T cell counts > = 100 cells/μl, the naïve/memory CD4+T cells would not transiently decrease at first three months (Figure 2).

### Dynamics of counts of CD8+ T cells and CD4/CD8 ratio

A significant decreased effect occurred on the absolute counts of CD8+ T cells during the first years. CD8+T cells decreased from 789 cells/μl (IQR, 532–1045) to 699 cells/μl (IQR, 527–925) after 3 years of treatment (Table 1). Hereafter, the CD8+T cell counts decreased slightly to 647 cells/μl (IQR, 515–843) after 10 years of ART. Baseline CD8+T cell counts of <500 cells/μl, 500–999 cells/μl, 1000–1499 cells/μl and >1500 cells/μl were 22.2%, 45.3%, 22.8% and 9.7% became 22.7%, 62.5%, 9.1% and 5.7% after 10-year of follow-up, respectively (The trends were seen in Figure 1D). In the mixed linear model, baseline CD8+T counts significantly influenced the CD8+T cells after ART. Higher CD4+T cell counts, delayed viral suppression may interfere with CD8+T cells descent. Patients who used ART in PI-based and INSTI-based achieved higher CD8+T cell counts than NNRTI-based patients after ART treatment (Table 3).

In our patients, CD4+/CD8+T ratio achieved from 0.25 (IQR 0.12–0.40) pre-treatment to 0.66 (IQR 0.41–0.92), 0.72 (IQR 0.47–1.02), 0.76 (IQR 0.57–1.04) after 3, 5, 10 years of ART, respectively (Table 1). The CD4/CD8 continued to be a marked year-over-year increase but maintained a substantial two-year increase until the seventh year and no significant increase until the tenth year (Figure 1E and Supplementary Table 3). In our mixed linear model, baseline CD8+T cells, baseline CD4+T cell counts were independent with CD4/CD8 ratio normalization, but the ART regimen did not (Table 3).

**Table 3:** The influencing factors of CD8+T cell, CD8+HLA-DR+T cell, CD8+CD38+T cell and CD4/CD8 dynamics with mixed linear model.

	**CD8+ T cell count**		**CD8+ HLA-DR+T cell%**		**CD8+CD38+ T cell%**		**CD4/CD8**	
	**Unadjusted Est. Coef (SE)^†^**	**Adjusted Est. Coef (SE)^†^**	**Unadjusted Est. Coef (SE)^†^**	**Adjusted Est. Coef (SE)^†^**	**Unadjusted Est. Coef (SE)^†^**	**Adjusted Est. Coef (SE)^†^**	**Unadjusted Est. Coef (SE)^†^**	**Adjusted Est. Coef (SE)^†^**
Age (years)								
35–49	−34 (25.9)	−45 (19.6)^‡^	5 (1.1)	5 (0.9)^‡^	−3 (1.2)	−4 (0.8)^‡^	0.01 (0.03)	0.07 (0.02)^‡^
>50	−39 (36.2)	−35 (27.4)	13 (1.6)	13 (1.3)^‡^	−2 (1.6)	−4 (1.1)^‡^	0.06 (0.04)	0.10 (0.03)^‡^
18–35	0				0			
Gender								
Female	−82 (35.7)	−17 (26.9)	−6 (1.6)	−5 (1.3)^‡^	0.6 (1.6)	2 (1.1)	−0.05 (0.04)	−0.016 (0.03)
Male	0		0		0			
CD4 count at baseline(cell/μl)								
50–100	35 (47.9)	−48 (36.2)	−0.3 (2.2)	3 (1.1)	−3 (2.2)	−2 (1.5)	0.09 (0.05)	0.085 (0.04)^‡^
100–200	−64 (38.2)	−162 (29.1)^‡^	4 (1.7)	7 (1.3)	−6 (1.8)	−6 (1.2)^‡^	0.22 (0.04)	0.23 (0.03)^‡^
>200	69 (31.1)	−218 (26.0)^‡^	4 (1.4)	7 (1.8)	−5 (1.4)	−7 (1.1)^‡^	0.44 (0.03)	0.49 (0.03)^‡^
<50	0		0		0			
Time after ART initiation (years)								
0.25	−36 (13.0)	−24 (14.3)	−9 (0.5)	−9 (0.5)^‡*^	−13 (0.6)	−13 (0.7)^‡*^	−0.29 (0.01)	0.17 (0.01)^‡*^
0.5	−91 (17.0)	−72 (19.3)^‡^	−16 (0.6)	−16 (0.7)^‡*^	−20 (0.8)	−20 (0.9)^‡*^	0.17 (0.01)	0.22 (0.01)^‡*^
1	−111 (19.2)	−88 (22.1)^‡^	−19 (0.7)	−19 (0.8)^‡^	−25 (0.9)	−25 (1.0)^‡*^	0.23 (0.01)	0.29 (0.01)^‡*^
2	−115 (20.8)	−88 (23.7)^‡^	−21 (0.8)	−21 (0.9)^‡^	−30 (0.9)	−29 (1.1)^‡*^	0.31 (0.01)	0.36 (0.01)^‡*^
3	−151 (22.6)	−121 (25.2)^‡^	−21 (0.8)	−21 (1.0)^‡^	−32 (1.0)	−32 (1.2)^‡^	0.39 (0.01)	0.42 (0.01)^‡*^
4	−184 (24.4)	−155 (26.4)^‡^	−23 (0.9)	−23 (1.0)^‡^	−34 (1.0)	−34 (1.2)^‡^	0.45 (0.01)	0.49 (0.01)^‡^
5	−186 (26.5)	−151 (28.0)^‡^	−22 (1.0)	−21 (1.2)^‡^	−36 (1.1)	−36 (1.3)^‡^	0.52 (0.02)	0.52 (0.02)^‡*^
6	−205 (28.1)	−167 (29.3)^‡^	−22 (1.1)	−21 (1.1)^‡^	−38 (1.1)	−38 (1.3)^‡^	0.54 (0.02)	0.58 (0.02)^‡*^
7	−220 (30.3)	−172 (31.1)^‡^	−22 (1.1)	−21 (1.2)^‡^	−39 (1.2)	−39 (1.4)^‡^	0.60 (0.02)	0.60 (0.02)^‡*^
8	−210 (33.2)	−154 (33.5)^‡^	−19 (1.3)	−18 (1.3)^‡*^	−42 (1.4)	−42 (1.5)^‡^	0.61 (0.02)	0.60 (0.02)^‡*^
9	−182 (37.7)	−123 (37.5)^‡^	−18 (1.4)	−17 (1.5)^‡*^	−44 (1.6)	−44 (1.7)^‡^	0.66 (0.03)	0.65 (0.02)^‡*^
10	−219 (43.7)	−155 (42.7)^‡^	−19 (1.6)	−18 (1.7)^‡*^	−43 (1.8)	−44 (1.9)^‡^	0.71 (0.03)	0.71 (0.03)^‡*^
0	0		0		0			
ART regimen								
2NRTIs+PI	219 (46.5)	144 (36.4)^‡^	4 (2.1)	2 (1.7)	5 (2.2)	3 (1.5)^‡^	0.03 (0.06)	NA
2NRTIs+INSTI	188 (40.5)	73 (32.4)^‡^	12 (1.8)	8 (1.5)^‡^	6 (1.9)	1 (1.4)	−0.09 (0.05)	NA
Others	185 (43.5)	38 (33.9)	−2 (2.0)	−3 (1.6)^‡^	4 (2.1)	3 (1.4)^‡^	−0.01 (0.06)	NA
2NRTIs+NNRTI	0							
Baseline CD8 counts								
500–999	162 (24.5)	232 (24.0)^‡^	3 (1.3)	3 (1.1)^‡^	−3.0 (1.4)	−1 (1.0)		−0.15 (0.02)^‡^
1000–1499	401 (28.5)	481 (28.5)^‡^	8 (1.6)	7 (1.3)^‡^	−2.9 (1.6)	−1 (1.2)		−0.30 (0.03)^‡^
>1500	789 (37.2)	864 (37.9)^‡^	9 (2.0)	7 (1.8)^‡^	−0.8 (2.1)	0 (1.6)		−0.35 (0.04)^‡^
<500	0		0		0			
VL <5 0 cps/ml								
No	−78 (12.3)	−31 (15.4)^‡^	−8 (0.5)	−0.8 (2.1)	−16 (0.6)	0 (0.7)	0.15 (0.01)	−0.075 (0.11)
Yes	0		0		0			

Abbreviations: ^†^Est. Coef.: estimated coefficient; SE.: standard error ^‡^Between group comparisons of the difference of values at particular time points and the baseline value, *P*-Value < 0.05; ^*^Adjusted multiple comparisons when compared with indicators at 5-year of ART, *P*-Value < 0.05.

### Other lymphocyte subpopulations

There was decreasing kinetics of CD38+CD8+/CD8+, HLA-DR+CD8+/CD8+T cells, increasing kinetics of CD28+CD4+/CD4+T cells and CD28+CD8+CD8+T cells after initiation of ART. The percentage of activated CD38+CD8+T cells pretreatment was 79.8% (IQR 68.5–89.3%). ART-induced viral suppression made the percentage decreased to 44.7% (IQR 32.1–57.3%), 41.6% (IQR 30.2–54.1%), 35.2% (IQR 27.2–44.7%), 31.0% (IQR 21.5–44.8%) after 3 year, 5 year, 8 year and 10 year (Table 1). The most dramatic change occurred within the 3 months of ART, and the major decrement of CD38+CD8+T cells % occurred during the 1–5 year of treatment (Table 3 and Supplementary Table 3). Baseline CD8+T cells was not significant with CD8+CD38+T cells, but baseline CD4+T cells were independent with CD8+CD38+T cells. ART regimens seem to have little effect on CD38+CD8+T cells kinetics, ART based on PI-backbone achieved higher CD38+ expression on CD8+T cells than NNRTI-backbone.

Activated HLA-DR+CD8+/CD8+ T cells at baseline in HIV-infected patients was 63.2% (IQR 48.3–74.2%) and it decreased to 34.9% (IQR 27.2–49.4%) after 10-years ART (Table 1). After one year of ART, the rapid decrease of HLA-DR+CD8+/CD8+T cells had no significant change between the next years. In the mixed linear model, older patients were higher than younger patients in related to percentage of HLA-DR+ CD8+T cells. Unlike the CD38+CD8+/CD8+ T cells, the percentage of HLA-DR+ expression on CD8+T cells was independently related with CD8+T cells instead of CD4+T cells. Delayed viral suppression had no significant effect on CD38+ expression on CD8+T cells and HLA-DR+ expression on CD8+T cells.

The expressing molecule CD28 on CD4+T cells and CD8+T cells were 89.2% (IQR 75.0–95.5%) and 30.5% (IQR 21.7–40.1%) before treatment, respectively. After 3-year of follow-up, the percentage inclined to 93.3% (IQR 86.5–97.4%), 48.5% (IQR 38.6, 60.1), respectively (Table 1). In our results, CD28+CD4+T cells significantly mounted within the second year, while CD28+CD8+T cells ascended significantly at the first five years after ART (seen in Supplementary Table 4).

Antiretroviral treatment and delayed viral suppression showed little influence on the CD28+ expression on CD4+ and CD8+T cells (seen in Supplementary Table 5). In patients with CD4>100 cells/μl pretreatment whose percentage of CD4+CD28+T cells increased significantly more than patients with CD4<100 cells/μl before treatment. Age and gender do not affect CD4+CD28+T cells but a significantly slower increase of CD8+CD28+T cells in older people and males. Higher CD4+T cell and lower CD8+T cell pretreatment contributed to a quicker climb of the CD8+CD28+/CD8+T cells.

## DISCUSSION

Our results have illustrated the tragedies of naive and memory subpopulation of CD4+T cells change for 10-year of follow-up with ART. We fitted the increasing trends of CD45RA+CD4+ and CD45RA-CD4+T cells at different time-point with real-world data. In addition, ten years of ART with undetectable viremia enabled the complete reconstitution of CD45RA+CD4+T cells and CD45RA-CD4+T cells, but significant change was observed at 6–8 years. In addition, the long-term viral suppression contributed to the decreased level HLA-DR+CD8+T cells and CD38+CD8+T cells, increased level of CD28+CD4+T cells and CD8+CD28+T cells to the levels of healthy controls after follow-up.

The ability to continuously reconstitute CD4+T cells count in HIV-infected patients during suppressive ART for longer periods is quite variable in literature data. Several longitudinal studies suggested that amount of CD4+T cells reconstitution for exceeding one or two years was restricted at the early era of the ART regimen [15, 16]. Slowly, the results of longer follow-up studies showed that CD4+T cell count in HIV-infected patients continuously ascended after virologically depressed even after 4–6 years of treatment, although there was a part of patients that could not continuously climb to a normal level if their CD4+T cell counts were lower pretreatment [6, 7, 9, 17, 18]. Until now, the longitudinal study followed more than 10 years conducted in Canada, found that 16% of patients achieved comprehensive T cell subsets reconstitution through a 10-year of sustained successful ART [19]. Although Dawit Wolday’s study suggested that 69.6% of patients achieved their amount of CD4+T cell normalization within 6.5 (IQR: 3.0–10.5) years. The CD4+T cell depicted in their study continuously climbed up till 5 years and plateaued until 14 years [20]. Compared with our research, the CD4+T cells would continuously increase until the seventh year of ART when we consider the baseline CD4+T cell counts, age, ART regimen, and virological suppression, which was consistent with the results of Dawit Wolday’s study [20] and other similar researches [6, 8, 21]. Interestingly, Studies in French [22] and ALLRT team [23] suggested that CD4+Tcell counts ascended in most patients for at least seven years if HIV-patients remained on ART, which is slightly different from our results since our results showed no significant change thereafter.

The kinetics of CD45RA-memory CD4+T cells and CD45RA+naïve CD4+T cells increased was mainly explained by a triphasic progress: an early surge with sharp slope followed by a gradual climbing, and subsequently string along with indolent growth [3]. The first two phases of CD4+T cells were mainly composed of CD4+memory T cells migration to the peripheral blood and followed by naive T cells refreshed from thymic. Therefore, with higher CD4+T cells at baseline, the number of memory T cells redistributing would be higher in HIV-infected patients. Thus, we also observed the significant change in memory CD4+T cell at first 3-month. CD45RA+Naïve CD4+T cells are often thought as a renewable and truly increased T cell from lymphoid organ. In the study [10] followed for four years, significant increase was observed in naive CD4+T cells at the second year after ART initiation. But there was little change of naïve CD4+T cells thereafter. In our results, CD45RA+ naïve CD4+T cells and CD45RA- memory CD4+T cells would be influenced by age, their change persisted until 4–6 years which was similar with CD4+T cell counts. The naïve/memory T cell ratio can be regarded as the competition of newly regeneration of T cells, which showed different model of recovery among groups of CD4<100 cells/μl and CD4>100/ cells/μl at baseline. This phenomenon can be partly explained by a severe degree of thymus damage in patients with CD4<100 cells/μl but homing CD4+T cell counts in peripheral lymphocyte organ than in patients with CD4>100 cells/μl. Besides, Longer ART treatment would lead to a more pronounced higher memory T cell and lower naïve T cell in HIV-infected patients than in healthy controls. Frederikke F Rönsholt [11] found lower percentage of Naive CD4 cells in 71 HIV-infected patients than in controls even after 12-year on ART, which was consistent with our study.

After persistent virus-related stimulation and activation and even inhibitor signal effect during HIV infection, CD28 expression on the CD8+T cells would downregulate. This phenomenon has been described previously and can be partly recovered through ART treatment in HIV-infected patients [10, 24]. Clinical literature reported that the complete reconstitution of CD4 and CD8 cells expressing CD28 required for 4-year from baseline but lower than healthy controls [24]. Similarly, a total of 1291 patients in chronic phrase followed 96-week found that removing covariance of age, sex, and race/ethnicity, the percentage of CD28 expression on both CD4+ and CD8+ T cell subsets was lower and abnormal than those of controls [25]. However, the percentage of CD28 expression on CD4+T cell in our research showed a rapid increase in the first year within the normal range at 2-year, while CD28 expression on CD8+T cell kept increased until the fifth year. CD8+CD28+T cells showed a trend of decrease with aging after ART in our results, which is in line with research showing that CD8+CD28−T cells are often regarded as symbols of short telomere length, promoting pro-inflammatory molecules production, and reducing proliferation signal [26, 27].

CD38 on CD8+ T lymphocytes as immune hyperactivation would make CD4+T cells more susceptible to HIV infection and contribute to HIV replication, which was proposed in the studies [12, 28]. Therefore, evaluating the expression of activating immune signatures, for example, CD38 (on CD8+T cells) is essential for assessment of comprehensive immune reconstitution in HIV-infected patients. Valdez et al. [24] have shown that abnormal CD8^+^ T cell activation in HIV-infected patients with CD4>100 cells/μl did not completely recover after 3-year of ART. Comparing with our study, higher percentage of CD38 on CD8+T cells have normalized after 4 years of ART in our patients. The major reduction in CD38+ overexpression occurred within the first 3 months of treatment. This result may be mainly explained by the significant suppression of plasma virus replication at the first 3 months [29]. Another activating immune signature marker HLA-DR expression on CD8+T cells was related to AIDS progression [30], which perform immune regulation in normal situation but exaggerated abnormal immune response in HIV-1 infection status [31]. In our data, a significant descend was seen in the HLA-DR+CD8+/CD8+% T cells after 1-year of ART. Whereas, no evident difference between time intervals after 1-year was observed in HIV-infected subjects regardless of their CD4+T cell counts pretreatment.

In general, our study showed longitudinal research of the kinetics of the T cell subsets reconstitution after 10-year ART. To our knowledge, this study provided largest sample size and longest time of T cell subpopulation change, which depicted the aging and the ART effect on the HIV-patients. Besides, we depicted the model of T cell subpopulation change over 10-years of ART with mixed linear model, considering the covariate effect including ART regimen and viral suppression as well as status at baseline, and comprehensively explained the data measured repeatedly.

Comparing with cohort in European and other developed countries, the limitation of our study covered up the following: firstly, it is the relatively small sample size after long-term visit. The predictive role of pattern and kinetics of CD4+T cell count and other T cell subpopulations will be clearer with more subjects. Another limitation was that first-line recommended ART regimens have changed during the followed time. Therefore, CD4+T cell trajectory and T cell subsets change might be influenced by starting a different ART regimen. As current ART can be well tolerated and effective in viral control because of reduced side effects or tablets leading to improved adherence. Last but not least, we confirmed that among these indicators, age has a significant effect on the T cell subpopulation, especially CD28, CD45RA expression, but this effect on the healthy people did not have longitudinal data as HIV-infected patients, which may partly explain the difference between our study with others.

Overall, our data showed longitudinal research of the kinetics of the reconstitution of CD4+T cells, CD8+T cells and CD4/CD8 ratio, naive CD45RA+CD4+T cells and memory CD45RA-CD4+T cells, CD28+ expression on CD4+T and CD8+T cells, and activation markers (CD38+ and HLA-DR expression on CD8+T cells) induced by ART. In our study, we found that most part of immune restoration was achieved within the 2–3 years after ART whatever the CD4+T or the other lymphocyte subpopulations, but slower change for next 3–4 years. The gap between the baseline status and the immune reconstitution at different time points was different, but almost all T cell subpopulation did not change significantly after 7–8 years when there counted nearly 100–200 patients on. Conclusively, Immune restoration after ART robustly within first 2–3 years already reflect the immune system response to whether achieve fully reconstitution.

## METHODS

### Patients

This cohort enrolled HIV–infected adults visiting the HIV/AIDS Clinic Center of Peking Union Medical College Hospital (PUMCH) and keeping follow-up since 2003. The cohort has been recruited for almost 20 years based on actual treatments, most of them ensuring regular follow-up. The criteria for enrollment were: (1) HIV-infection based on serology and HIV-1 RNA, (2) ART-naive, (3) age 18–65, (4) both genders (4) with regular follow-up documents. Patients with an AIDS-defining disease or CD4+T cell counts below 200 cells/μl were initiated with a combination of antiretroviral therapy prior to 2015, in accordance with national HIV/AIDS treatment guidelines. After 2015, if HIV infection was confirmed, patients were recommended for therapy. After meeting these criteria, we retrospectively recruited 662 HIV-infected patients followed by at least 2 years who had eliminated the HIV-1 RNA plasma load <50 copies/ml. The flow chart of participants selection was seen in Supplementary Figure 1. The study was approved by an independent ethics committee and the institutional review board of PUMCH (Peking Union Medical College Hospital, JS-1431). The trial was carried out in accordance with the principles of Good Clinical Practice and the Declaration of Helsinki. Written informed consent was obtained from all the participants.

Main laboratory analysis (including T cell subsets and HIV viral load) were conducted at baseline, followed by 3 months, 6 months, 12 months, and every 6 months thereafter. Plasma samples for laboratory tests were taken during routine diagnosis.

### Laboratory test

In order to assess the T cell immune response in HIV infected patients, we compared lymphocyte subpopulations in subjects before and during treatment to reference range, which examined 56 healthy blood donors (36 male, mean age was 36 ± 10 years old) in PUMCH and established reference value range since 2002 [32, 33]. Immune profiles were analyzed by three-color flow cytometry (Epics XL flow cytometry; Beckman Coulter, USA) as previously described [34]. Freshly EDTA-anticoagulated whole blood was incubated, lysed, and tested. The combination of monoclonal antibodies panels is as following: CD3/CD8/CD4, CD3/CD16CD56/ CD19, HLA-DR/CD38/CD8, CD28/CD8/CD4, CD45RA/CD4 and isotype controls (Immunotech, France). By measuring white blood cell counts and lymphocyte differentials from routine blood tests, cell counts of lymphocyte subsets were calculated of the same sample. HIV-RNA plasma viral load was examined by COBAS Ampliprep/TaqMan 48 RT-PCR real-time test (Roche, CA, USA).

### Statistical analysis

Kolmogorov-Smirnov test was used to examine data distribution. The mean standard deviation with mean were used for parametric data and 25% and 75% for non-parametric data. two groups and parametric data comparison need student-test, non-parametric data demands for Mann-Whit test, *χ*^2^ test for categorical variables.

Analysis of lymphocyte subsets was performed in individuals with an initial CD4+T count and available CD4+T cell records for at least twice visits. The linear mixed model (MIXED) with repeated measurements intended for identifying the possible covariates and time effect on repeated measurements of the immune profile. The mixed linear model was used to represent T cell subpopulation trajectories. Let *y_it_* (*i* = 1, 2, … , *n*; *t* = 1, 2, … , *ni*) be the immune indicators of HIV infected patient *i* at follow-up visit time *t*. The longitudinal immune indicators dynamics *y_it_* depicted the following model:


$$y_{i}\text{(}t\text{)}=x_{i}^{\prime }\text{(}t\text{)}\beta +z_{i}^{\prime }\text{(}t\text{)}b_{i}+\varepsilon_{i}\text{(}t\text{)}$$

where $x_{i}^{\prime }\text{(}t\text{)}$ and $z_{i}^{\prime }\text{(}t\text{)}$ represents time-varying covariates with fixed effects β and random effects *b_i_*, respectively. Random effects represent the effects of each subject that cannot be explained by the observed covariates. The errors in our assumption are independent and follow a normal distribution, *ε_i_*(*t*) ~N(0, σ), and that *b_i_* and *ε_i_*(*t*) are independent. The unstructured covariance of fixed effect was assumed for the covariance structure. Model fit statistics (e.g., AIC, BIC, etc) were used to choose the best covariance structure. To check the effect of time on CD4, we used the Scheffe-Test as post-hoc comparison. *P*-value < 0.05 (two-side) was regarded as significant.

Statistical analysis was performed with SAS software version 9.4 and SPSS software (SPSS^®^ for Windows^™^ version 22.0, SPSS Inc., Chicago, IL, USA). Plots were drawn by GraphPad Prism 8.

## Supplementary Materials

Supplementary Figure 1

Supplementary Tables

## Abbreviations

HIV-1: human immunodeficiency virus type 1
ART: antiretroviral therapy
NRTIs: Nucleoside reverse-transcriptase inhibitors
NNRTI: Non-Nucleoside reverse-transcriptase inhibitors
PI: Proteinase inhibitor
INSTI: Integrase inhibitor
