# Supplementary figures and images for: The pattern and magnitude of T cell subsets reconstitution during ten years of ART with viral suppression in HIV-infected patients
Source: Aging (Albany NY). 2022 Dec 9;14(23):9647–67. doi: 10.18632/aging.204416 (PMC9792206; doi:10.18632/aging.204416)

## SUPPLEMENTARY FIGURE

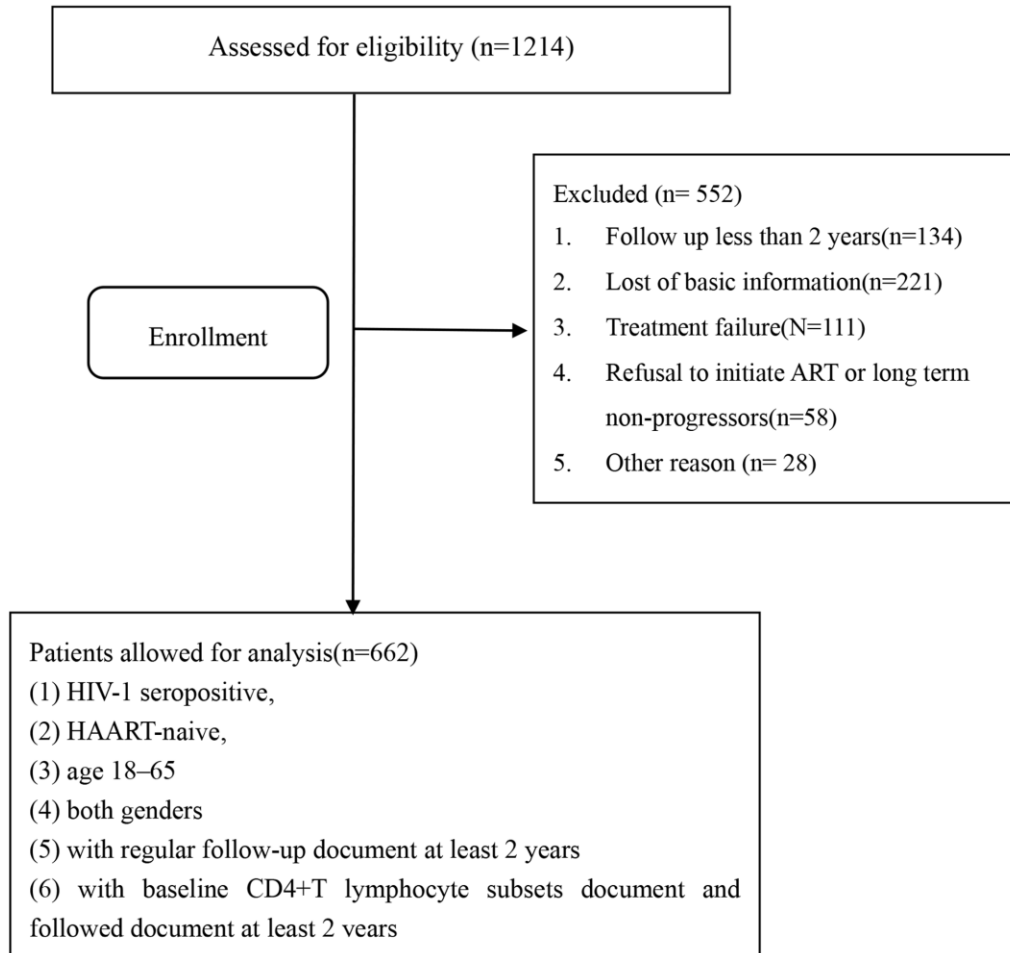

**Supplementary Figure 1. Flow chart of patients in enrollment.**

Supplement: Supplementary Figure 1 [file aging-14-204416-s001.pdf]
