# Supplementary material for: The pattern and magnitude of T cell subsets reconstitution during ten years of ART with viral suppression in HIV-infected patients
Source: Aging (Albany NY). 2022 Dec 9;14(23):9647–67. doi: 10.18632/aging.204416 (PMC9792206; doi:10.18632/aging.204416)
Supplement: Supplementary Tables [file aging-14-204416-s002.pdf]

SUPPLEMENTARY TABLES

Supplementary Table 1. Adjusted multiple comparisons of CD3+CD4+T cell counts between different time-point after ART treatment (SCHEFFE comparison).

Time after ART	0.25	0.5	1	2	3	4	5	6	7	8	9	10	CD4+T cell count (cell//μl)‡
0.25	NA												347
0.5	0.0115	NA											373
1	<.0001	<.0001	NA										407
2	<.0001	<.0001	<.0001	NA									463
3	<.0001	<.0001	<.0001	0.0018	NA								487
4	<.0001	<.0001	<.0001	<.0001	**0.3021**†	NA							498
5	<.0001	<.0001	<.0001	<.0001	0.0117	**0.7113**	NA						512
6	<.0001	<.0001	<.0001	<.0001	<.0001	0.015	**0.4775**	NA					522
7	<.0001	<.0001	<.0001	<.0001	<.0001	0.0236	**0.5578**	**1**	NA				519
8	<.0001	<.0001	<.0001	<.0001	<.0001	<.0001	0.0216	**0.6491**	**0.7629**	NA			529
9	<.0001	<.0001	<.0001	<.0001	<.0001	<.0001	<.0001	0.0281	**0.0596**	**0.7079**	NA		548
10	<.0001	<.0001	<.0001	<.0001	<.0001	<.0001	0.0031	**0.1546**	**0.2958**	**0.9452**	**1**	NA	554
0	<.0001	<.0001	<.0001	<.0001	<.0001	<.0001	<.0001	<.0001	<.0001	<.0001	<.0001	<.0001	237

‡CD4+T cell counts: the CD4+T-cell count predicted from mixed linear model. Bold characters showed no significance among adjusted comparisons.

Supplementary Table 2. Adjusted multiple comparisons of CD4+CD45RA- and CD4+CD45RA+T cell counts between different time-point after ART treatment (SCHEFFE comparison).

Time after ART	0.25	0.5	1	2	3	4	5	6	7	8	9	10	CD4+CD45RA-T (cells//µl)‡
0.25	NA												232
0.5	**0.1161**	NA											248
1	<.0001	<.0001	NA										270
2	<.0001	<.0001	<.0001	NA									306
3	<.0001	<.0001	<.0001	**0.1423**	NA								318
4	<.0001	<.0001	<.0001	0.0002	**0.2922**	NA							333
5	<.0001	<.0001	<.0001	<.0001	0.0002	**0.1202**	NA						354
6	<.0001	<.0001	<.0001	<.0001	<.0001	0.0004	**0.4785**	NA					364
7	<.0001	<.0001	<.0001	<.0001	<.0001	0.027	**0.9416**	**1**	NA				363
8	<.0001	<.0001	<.0001	<.0001	<.0001	<.0001	**0.1251**	**0.9412**	**0.7282**	NA			380
9	<.0001	<.0001	<.0001	<.0001	<.0001	<.0001	0.0103	**0.4154**	**0.2477**	**0.9761**	NA		403
10	<.0001	<.0001	<.0001	<.0001	<.0001	<.0001	0.0091	**0.2899**	**0.2106**	**0.9196**	**1**	NA	420
0	<.0001	<.0001	<.0001	<.0001	<.0001	<.0001	<.0001	<.0001	<.0001	<.0001	<.0001	<.0001	160
													**CD4+CD45RA+T (cells//µl)‡**
0.25	NA												115
0.5	0.0024	NA											124
1	<.0001	<.0001	NA										136
2	<.0001	<.0001	<.0001	NA									159
3	<.0001	<.0001	<.0001	<.0001	NA								163
4	<.0001	<.0001	<.0001	<.0001	0.0404	NA							163
5	<.0001	<.0001	<.0001	<.0001	**0.0914**	**0.8218**	NA						164
6	<.0001	<.0001	<.0001	<.0001	0.0283	**0.3279**	**0.3286**	NA					161
7	<.0001	<.0001	<.0001	<.0001	0.0102	**0.1371**	**0.15**	**0.3871**	NA				160
8	<.0001	<.0001	<.0001	<.0001	0.0001	0.0045	0.0054	0.0189	0.0441	NA			160
9	<.0001	<.0001	<.0001	<.0001	<.0001	0.0004	0.0006	0.0025	0.0081	**0.1585**	NA		168
10	<.0001	<.0001	<.0001	<.0001	0.0059	0.0446	**0.0557**	**0.1326**	**0.2712**	**0.9538**	**0.2172**	NA	150
0	<.0001	<.0001	<.0001	<.0001	<.0001	<.0001	<.0001	<.0001	<.0001	<.0001	<.0001	<.0001	78

‡CD4+CD45RA- and CD4+CD45RA+T: the CD4+CD45RA- and CD4+CD45RA+T-cell predicted from mixed linear model. Bold characters showed no significance among adjusted comparisons.

Supplementary Table 3. Adjusted multiple comparisons of other T-cell subsets between different time-point after ART treatment (SCHEFFE comparison).

Time after ART	0.25	0.5	1	2	3	4	5	6	7	8	9	10	CD8+HLA-DR+T cell (%)‡
0.25	NA												51.34
0.5	<.0001	NA											44.49
1	<.0001	<.0001	NA										41.18
2	<.0001	<.0001	**0.1841**	NA									39.22
3	<.0001	<.0001	**0.8619**	**1**	NA								39.04
4	<.0001	<.0001	**0.1446**	**0.9926**	**0.8696**	NA							37.11
5	<.0001	0.0008	**0.9235**	**1**	**1**	**0.9964**	NA						37.82
6	<.0001	0.006	**0.9738**	**1**	**1**	**0.9994**	**1**	NA					37.65
7	<.0001	**0.0648**	**0.9985**	**1**	**1**	**0.9982**	**1**	**1**	NA				37.74
8	<.0001	**0.9987**	**0.9999**	**0.7692**	**0.8348**	**0.1702**	**0.5905**	**0.5065**	**0.4242**	NA			41.07
9	0.0019	**1**	**0.9977**	**0.6695**	**0.7448**	**0.1747**	**0.5545**	**0.5443**	**0.6249**	**1**	NA		41.42
10	0.004	**0.9999**	**1**	**0.9752**	**0.986**	**0.7459**	**0.9589**	**0.9646**	**0.9854**	**1**	**1**	NA	40.69
0	<.0001	<.0001	<.0001	<.0001	<.0001	<.0001	<.0001	<.0001	<.0001	<.0001	<.0001	<.0001	60.51
													**CD8+CD38+T cell (%)‡**
0.25	NA												63.64
0.5	<.0001	NA											56.97
1	<.0001	<.0001	NA										51.76
2	<.0001	<.0001	<.0001	NA									47.51
3	<.0001	<.0001	<.0001	**0.5776**	NA								45.17
4	<.0001	<.0001	<.0001	0.0037	**0.5066**	NA							42.97
5	<.0001	<.0001	<.0001	<.0001	**0.1031**	**0.9831**	NA						41.33
6	<.0001	<.0001	<.0001	<.0001	0.0003	**0.2473**	**0.8703**	NA					39.03
7	<.0001	<.0001	<.0001	<.0001	<.0001	**0.1186**	**0.7336**	**1**	NA				38.01
8	<.0001	<.0001	<.0001	<.0001	<.0001	0.0008	**0.0562**	**0.7379**	**0.9312**	NA			35.04
9	<.0001	<.0001	<.0001	<.0001	<.0001	0.0002	0.016	**0.4174**	**0.7514**	**0.9998**	NA		33.81
10	<.0001	<.0001	<.0001	<.0001	<.0001	0.0049	**0.0901**	**0.6713**	**0.9091**	**1**	**1**	NA	33.98
0	<.0001	<.0001	<.0001	<.0001	<.0001	<.0001	<.0001	<.0001	<.0001	<.0001	<.0001	<.0001	77.24
													**CD4+CD28+T cell (%)‡**
0.25	NA												86.23
0.5	**0.9971**	NA											86.86
1	**0.3178**	**0.6785**	NA										87.96
2	<.0001	0.0001	0.0179	NA									89.64
3	<.0001	<.0001	0.0041	**0.9601**	NA								90.46
4	<.0001	<.0001	0.0003	**0.491**	**0.9682**	NA							91.06
5	<.0001	<.0001	0.0114	**0.854**	**0.9993**	**1**	NA						90.93
6	<.0001	<.0001	**0.0057**	**0.6616**	**0.9841**	**1**	**1**	NA					91.19
7	<.0001	<.0001	0.0073	**0.5966**	**0.9629**	**1**	**0.9997**	**1**	NA				91.2
8	0.0003	0.0034	**0.0895**	**0.8993**	**0.998**	**1**	**1**	**1**	**1**	NA			90.44

9	<.0001	0.001	0.0276	**0.6027**	**0.9312**	**0.9987**	**0.9977**	**0.9999**	**1**	**0.9996**	NA		91.28
10	0.0159	**0.0582**	**0.3197**	**0.9451**	**0.9976**	**1**	**1**	**1**	**1**	**1**	**1**	NA	90.06
0	<.0001	<.0001	<.0001	<.0001	<.0001	<.0001	<.0001	<.0001	<.0001	<.0001	<.0001	<.0001	80.74
													**CD8+CD28+T cell (%)‡**
0.25	NA												37.63
0.5	<.0001	NA											40.01
1	<.0001	0.0024	NA										42.05
2	<.0001	<.0001	<.0001	NA									45.78
3	<.0001	<.0001	<.0001	<.0001	NA								49.44
4	<.0001	<.0001	<.0001	<.0001	<.0001	NA							51.99
5	<.0001	<.0001	<.0001	<.0001	<.0001	**0.981**	NA						52.89
6	<.0001	<.0001	<.0001	<.0001	<.0001	**0.2978**	**0.8224**	NA					54.27
7	<.0001	<.0001	<.0001	<.0001	<.0001	**0.1155**	**0.5744**	**0.9989**	NA				54.86
8	<.0001	<.0001	<.0001	<.0001	<.0001	**0.0802**	**0.4678**	**0.988**	**1**	NA			54.86
9	<.0001	<.0001	<.0001	<.0001	<.0001	0.0417	**0.288**	**0.9083**	**0.996**	**0.9999**	NA		55.46
10	<.0001	<.0001	<.0001	<.0001	<.0001	**0.106**	**0.4223**	**0.9287**	**0.9957**	**0.9998**	**1**	NA	55.02
0	<.0001	<.0001	<.0001	<.0001	<.0001	<.0001	<.0001	<.0001	<.0001	<.0001	<.0001	<.0001	31.69

CD8+HLA-DR+T cell (%), CD8+CD38+T cell (%), CD4+CD28+T cell (%), CD8+CD28+T cell (%). ‡These T-cell subsets predicted from mixed linear model. Bold characters showed no significance among adjusted comparisons.

Supplementary Table 4. Adjusted multiple comparisons of CD3+CD8+T cell counts and CD4/CD8 ratio between different time-point after ART treatment (SCHEFFE comparison).

Time after ART	0.25	0.5	1	2	3	4	5	6	7	8	9	10	CD3+CD8+T (cells//μl)‡
0.25	NA												880
0.5	**0.4127**	NA											825
1	**0.3736**	**0.9999**	NA										803
2	**0.584**	**1**	**1**	NA									799.
3	**0.0729**	**0.9086**	**0.9897**	**0.9507**	NA								757
4	0.0018	**0.2701**	**0.5399**	**0.3967**	**0.9686**	NA							730
5	0.0137	**0.5356**	**0.8133**	**0.776**	**0.9993**	**1**	NA						730
6	0.0045	**0.3072**	**0.5891**	**0.5601**	**0.9871**	**1**	**1**	NA					699
7	0.0089	**0.3595**	**0.6347**	**0.6234**	**0.9883**	**1**	**1**	**1**	NA				681
8	**0.1421**	**0.8388**	**0.9601**	**0.9601**	**1**	**1**	**1**	**1**	**1**	NA			699
9	**0.8057**	**0.9992**	**1**	**1**	**1**	**1**	**1**	**0.9997**	**0.9989**	**1**	NA		723
10	**0.596**	**0.9785**	**0.9965**	**0.9966**	**1**	**1**	**1**	**1**	**1**	**1**	**1**	NA	694
0	**0.9964**	**0.2989**	**0.204**	**0.3165**	0.0281	0.0007	0.0041	0.0013	0.0025	**0.0508**	**0.5558**	**0.3576**	916
													**CD4+/CD8+T‡**
0.25	NA												0.46
0.5	<.0001	NA											0.52
1	<.0001	<.0001	NA										0.60
2	<.0001	<.0001	<.0001	NA									0.68

3	<.0001	<.0001	<.0001	<.0001	NA								0.73
4	<.0001	<.0001	<.0001	<.0001	<.0001	NA							0.78
5	<.0001	<.0001	<.0001	<.0001	<.0001	**0.6465**	NA						0.80
6	<.0001	<.0001	<.0001	<.0001	<.0001	<.0001	0.0022	NA					0.85
7	<.0001	<.0001	<.0001	<.0001	<.0001	<.0001	0.0147	**0.9999**	NA				0.85
8	<.0001	<.0001	<.0001	<.0001	<.0001	0.0008	**0.0879**	**0.9999**	**1**	NA			0.83
9	<.0001	<.0001	<.0001	<.0001	<.0001	<.0001	0.0005	**0.5187**	**0.7859**	**0.6482**	NA		0.87
10	<.0001	<.0001	<.0001	<.0001	<.0001	<.0001	<.0001	0.0186	**0.0626**	**0.0434**	**0.7215**	NA	0.89
0	<.0001	<.0001	<.0001	<.0001	<.0001	<.0001	<.0001	<.0001	<.0001	<.0001	<.0001	<.0001	0.29

‡CD3+CD8+T cell counts and CD4/CD8 ratio: the CD3+CD8+T cell counts and CD4/CD8 ratio predicted from mixed linear model. Bold characters showed no significance among adjusted comparisons.

Supplementary Table 5. The influencing factors of percentage of CD4+ CD28+T cell and CD8+CD28+ T cell dynamics with mixed linear model.

	CD4+ CD28+T cell		CD8+CD28+ T cell	
	Unadjusted Est. Coef (Std. Err.)†	Adjusted Est. Coef (Std. Err.)†	Unadjusted Est. Coef (Std. Err.)†	Adjusted Est. Coef (Std. Err.)†
Age				
35–49	−2.1 (0.9)	−1.0 (0.8)	−4.1 (1.1)	−3.0 (0.8)‡
>50	−3.0 (1.3)	−2.1 (1.2)	−9.8 (1.5)	−9.4 (1.2)‡
18–35	0		0	
Gender				
Female	0.4 (1.3)	0.9 (1.2)	−1.9 (1.5)	−2.5 (1.1)‡
Male	0		0	
CD4 count at baseline (cells//µl)				
50–100	−2.4 (1.6)	−2.2 (1.5)	1.0 (2.0)	3.2 (1.5)‡
100–200	3.0 (1.3)	3.3 (1.2)‡	3.4 (1.6)	5.6 (1.2)‡
>200	7.8 (1.0)	8.3 (1.1)‡	6.9 (1.3)	11.4 (1.1)‡
<50	0		0	
Baseline CD8 counts (cells//µl)				
500–999	1.5 (1.1)	NA	0.4 (1.3)	−3.1 (1.0)‡
1000–1499	1.6 (1.2)	NA	−3.2 (1.5)	−8.0 (1.2)‡
>1500	2.1 (1.6)	NA	−3.2 (1.9)	−9.5 (1.6)‡
<500	0		0	
Time after ART initiation (years)				
0.25	5.5 (0.3)	5.4 (0.3)‡*	5.9 (0.3)	6.0 (0.4)‡*
0.5	6.0 (0.4)	6.0 (0.4)‡*	8.3 (0.5)	8.3 (0.5)‡*
1	7.1 (0.5)	7.1 (0.5)‡*	10.2 (0.5)	10.3 (0.5)‡*
2	8.8 (0.5)	8.8 (0.5)‡	13.9 (0.6)	14.0 (0.5)‡*
3	9.6 (0.6)	9.7 (0.6)‡	17.7 (0.6)	17.7 (0.6)‡*
4	10.5 (0.6)	10.6 (0.6)‡	20.5 (0.7)	20.6 (0.7)‡*
5	10.4 (0.7)	10.5 (0.7)‡	21.4 (0.8)	21.6 (0.7)‡

6	10.8 (0.8)	11.0 (0.8)‡	22.8 (0.8)	23.0 (0.8)‡
7	11.1 (0.9)	11.4 (0.8)‡	23.6 (0.9)	23.9 (0.9)‡
8	10.6 (0.9)	11.1 (0.9)‡	24.0 (1.0)	24.6 (0.9)‡
9	11.4 (1.1)	12.1 (1.1)‡	24.7 (1.1)	25.5 (1.1)‡
10	10.5 (1.3)	11.6 (1.2)‡	24.8 (1.3)	25.9 (1.3)‡
0	0		0	
ART regimen				
2NRTIs+INSTI	−0.6 (1.6)	NA	−0.6 (2.0)	2.7 (1.5)
2NRTIs+PI	−2.7 (1.4)	NA	−5.2 (1.7)	0.8 (1.3)
Others	−0.4 (1.6)	NA	−1.9 (1.9)	−0.02 (1.4)
2NRTIs+NNRTI	0		0	
VL <50 cps/ml				
No	7.7 (5.3)	NA	7.6 (6.4)	NA
Yes	0			

Abbreviations: †Est. Coef.: estimated coefficient; Std. Err.: standard error. ‡Between group comparisons of the difference of values at particular time points and the baseline value, *P*-Value < 0.05; *Adjusted multiple comparisons when compared with indicators at 5-year of ART, *P*-Value < 0.05.
